# Source-level motion artifact suppression in wearable electrodes: From underlying mechanisms to advanced strategies

**DOI:** 10.1016/j.mtbio.2026.103241

**Published:** 2026-05-16

**Authors:** Haizhou Huang, Xuanjing Cai, Shu Wan, Litao Sun

**Affiliations:** a College of Photonic and Electronic Engineering, Fujian Normal University, Fuzhou, 350117, PR China; b Key Laboratory of Optoelectronic Technology and Systems, Ministry of Education, Key Disciplines Laboratory of Novel Micro-Nano Devices and System Technology, School of Optoelectronics Engineering, Chongqing University, Chongqing, 400044, PR China; c SEU-FEI Nano-Pico Center, Key Laboratory of MEMS of Ministry of Education, Collaborative Innovation Center for Micro/Nano Fabrication, Device and System, Southeast University, Nanjing, 210096, PR China

**Keywords:** Motion artifact, Electrophysiological signals, Electrodes, Motion noise removal

## Abstract

Motion artifacts (MAs) significantly degrade the quality of surface biopotential signals acquired in dynamic environments (such as during body movements), severely compromising their reliable application in health monitoring and disease diagnosis. Although various post-processing methods have been reported to mitigate MAs, they often entail inherent limitations (such as signal distortion, latency, and incomplete artifact removal), highlighting the necessity of suppressing MAs at their source. This review provides an in-depth analysis of the mechanisms underlying MA generation and systematically summarizes source-level MAs suppression strategies based on innovations in electrode materials and structures. These strategies mainly include skin-anchoring strategy, deformation constraint strategy, strain-insensitive strategy and damping strategy. By stabilizing the skin–electrode interface and mitigating mechanical interference, they significantly reduce the occurrence of artifacts. The advantages and challenges of these strategies are further evaluating and future prospects toward achieving motion-artifact-free biopotential dynamic recording are also discussed. This review offers a clear framework and direction for the design of high-performance electrophysiological electrodes tailored for dynamic applications.

## Graphical abstract

**Figure:**
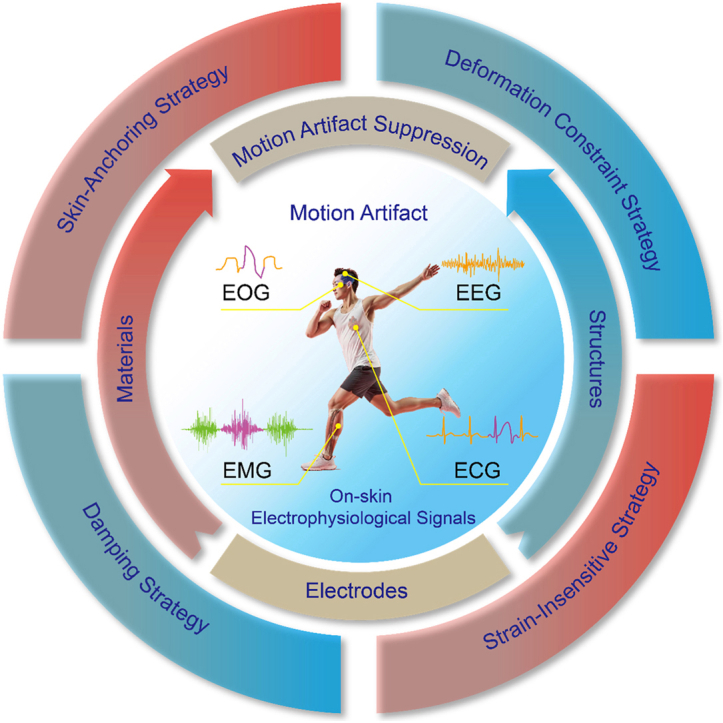

## 1 Introduction

Electrophysiological (EP) signals, such as electrocardiogram (ECG), electromyogram (EMG), electrooculogram (EOG) and electroencephalogram (EEG), are weak biopotential signals but they serve as vital physiological indicators of humans [1]. They can provide insights into the physiological functional states of interior tissues or organs through bioelectrical activities. Precise acquisition these EP signals are indispensable in clinical diagnosis, human health monitoring, human-machine interactions (HMI) and brain-computer interfaces (BCI) [2,3]. For instance, ECG captures myocardial conduction patterns critical for detecting arrhythmias, while EMG records electrical activities of muscle essential for diagnosing neuromuscular disorders and muscular diseases. Moreover, EP signals acquired during physical exercise can guide patient rehabilitation and activity regimens [4].

However, the transition of EP monitoring technologies from controlled clinical settings to real-world dynamic scenarios, is severely hampered by a persistent challenge: MAs. MAs is commonly defined as undesirable temporary voltage distortions caused by body movements during EP acquisition [5]. MAs can overwhelm or even exceed the ultralow-amplitude EP signals (typically 5 μV–10 mV) by several orders of magnitude, leading to erroneous data interpretation and significant risks of misdiagnosis [6]. Conventional countermeasures require subjects to remain still or even hold their breath during EP signal acquisition, limiting applications to static clinical contexts such as hospitals and laboratories [7,8]. Such constraints are particularly problematic in real-world scenarios where human motions (*e.g.*, breathing, walking, running and performing other daily activities), inevitably introducing MAs into EP recordings [5].

To mitigate MAs, conventional approaches predominantly rely on post-processing strategies, including signal processing techniques (*e.g.*, adaptive filtering, discrete wavelet transform), filtering algorithms (*e.g.*, Butterworth filters, finite impulse response) and machine learning [9]. These strategies operate after MAs have occurred, acting as a remedial measure after MAs generation. Although many progress has been made, they are inherently limited by the trade-off between noise removal and signal distortion (*e.g.*, useful information lost), particularly when the artifact and physiological signal share overlapping frequency bands (0.1‒30 Hz) [10]. Moreover, their computational complexity and high latency render them unsuitable for real-time, long-term EP monitoring applications [5]. These inherent drawbacks of post-processing strategies highlight the urgent need for a paradigm shift towards innovative source-level suppression approaches that prevent the generation of MAs from the source.

"Electrode design" is the key to addressing such MAs issue from the source, particularly materials and structures that interface with the skin [11]. As the fundamental element for capturing EP signals [12], electrodes convert ionic currents into measurable voltages [13]. Conventional rigid metal dry electrodes are highly susceptible to MAs due to mechanical mismatch with soft human skin and unstable contact during motions [14]. Similarly, although Ag/AgCl gel electrodes are regarded as the gold standards in clinical settings, they are also sensitive to MAs and exhibit shortcomings, including skin irritation and gel dehydration after prolonged use. As promising alternatives, recent advances in flexible on-skin electrode designs have demonstrated the capability to suppress MAs in dynamic environments by stabilizing skin-electrode interfaces and minimizing mechanical disturbances [15]. These advancements offer unparalleled advantages over post-processing strategies by preventing the generation of MAs in advance, thus preserving signal integrity in dynamic conditions.

**Fig. 1:**
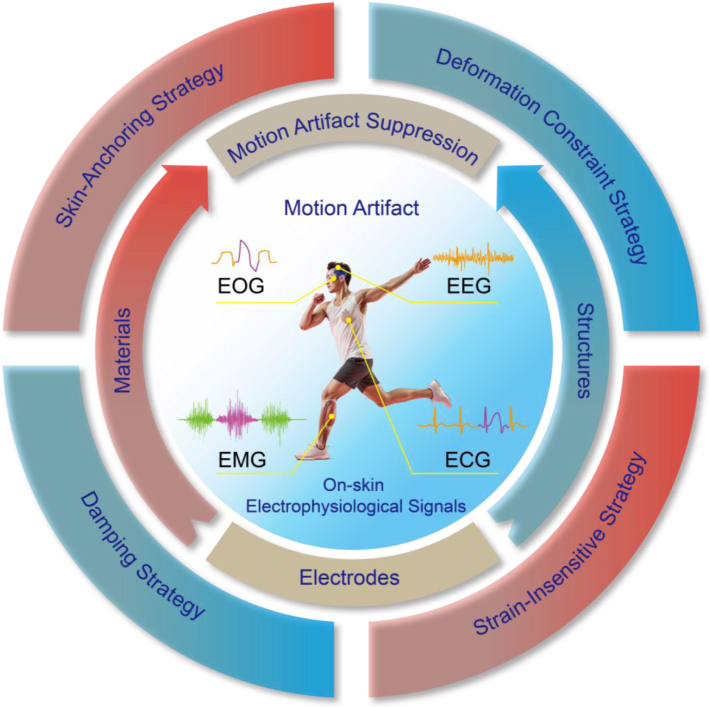
Motion artifacts of EP signals and its source-level suppression strategies.

In contrast to existing encyclopedic reviews that broadly cover MAs in bioelectronics [9,15], the strategies discussed therein are not always adequate for addressing MAs in the specific context of electrophysiological electrodes. Therefore, this review provides a more focused and in-depth perspective, which specifically aims to establish a strategic framework for the source-level management of MAs in electrophysiological electrodes, with an emphasis on innovations in electrode materials and structural design. While prior reviews have explored relevant material and structural advancements in electrodes [12,16], their discussions of MAs remain cursory and lack systematicity. This review fills this gap by comprehensively analyzing emerging source-level MAs suppression strategies tailored for dynamic EP measurements (Fig. 1). This review begins by outlining the significance of source-level MA suppression in EP signals and establishing fundamental knowledge of major EP signals (ECG, EMG, EEG, EOG). Subsequently, the physiological and mechanical origins of MAs through electrode-skin interface analysis are elucidated. Based on these generation mechanisms of MAs, recent advancements in source-level strategies have been presented, including: 1) skin-anchoring to enhance interfacial stability by minimizing the relative movements; 2) deformation constraint designs to restrict epidermal deformation; 3) strain-insensitive designs to minimize the impact of strain applied on the electrodes; and 4) viscoelastic damping mechanisms to dissipate mechanical energy. Finally, the review concludes with a discussion of challenges and future prospects toward MA-free dynamic EP recording.

## 2 Typical electrophysiological signals

EP signals, the low-amplitude bioelectrical potentials typically generated by electrical activities of excitable cells within nervous and muscle systems [12,17], serve as crucial indicators for assessing physiological functions and health status. The fundamental insights into excitable cells were initially established by Hodgkin and Huxley, who explained how cell membrane potentials are generated and altered [18,19].

The generation of EP signals relies on the differences in ion concentrations distribution between the interior and exterior of cell membrane during physiological processes, leading to a potential difference across the membrane [20]. In the resting state (no external stimuli), the cell exterior maintains a positive charge while the interior is a negative charge, resulting in a resting potential. When external stimuli exceed the excitation threshold, excitable cells activate, triggering the opening of Na^+^ and K^+^ ion channels. This allows Na^+^ influx and K^+^ efflux through the ion-selective permeable cell membrane. This rapid charge shift (occurring in millisecond) reverses the membrane polarity (negative exterior, positive interior). This depolarization process generates an action potential (AP). Following AP propagation to adjacent cells, Na^+^ and K^+^ ion pumps restore the original ion distribution, gradually repolarizing the membrane potential to its resting state. Repetitive depolarization-repolarization cycles generate rhythmic fluctuations in cellular membrane potential.

When groups of excitable cells are synchronously activated, their combined electrical activities create a comprehensive bioelectric field (potential) within nerve or muscle tissue, which finally manifest as measurable EP signals via the conduction of tissues and body fluids to the body surface. By placing electrodes on the targeted skin surfaces, these weak EP signals (*e.g.*, ECG, sEMG, EEG, or EOG) can be acquired, amplified and filtered for further analysis. The following subsections introduce fundamental characteristics of primary EP signals (ECG, EMG, EEG, and EOG) and their significance in physiological monitoring.

### 2.1 ECG

ECG directly reflects the events during myocardial conduction process, recording real-time changes in the electrical activity of the heart during each cardiac cycle [12]. As a critical non-invasive tool, ECG signals are extensively utilized for diagnosing cardiac function and enabling early detection of abnormalities (*e.g.*, arrhythmias, myocardial infarction, heart failure, sudden cardiac arrest) [21,22]. Besides, features derived from ECG (*e.g.*, heart rate variability), provide valuable insights for evaluating physical/mental fatigue, stress levels, and recovery status [23].

**Fig. 2:**
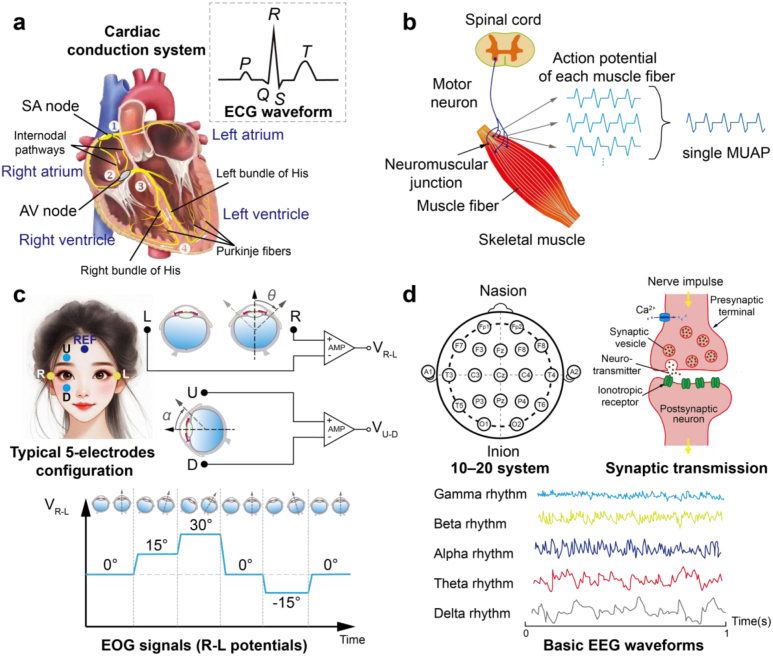
Origins of different EP signals and their characteristics. (a) Schematic diagram of the cardiac electrical conduction system in the heart along with the typical waveform and features of ECG signals. The numbers and arrows represent the pathways of cardiac electrical activity conduction. (b) Schematic diagram of the generation of EMG and action potentials from single motor unit. (c) Typical electrodes configuration for EOG recording and the mechanism of EOG signal generation. And EOG signal waveforms measure from the potential difference between the L and R electrodes (bottom). (d) Electrode locations of the international 10–20 system for EEG recording (upper left). Neurons communicate with each other at the synapse (upper right). When an action potential reaches the presynaptic terminal, it causes the release of neurotransmitter vesicles into the synaptic cleft. These neurotransmitters then diffuse across the cleft to bind with membrane receptors on the postsynaptic terminal, resulting in the generation of an excitatory postsynaptic potential. Basic EEG waveforms (bottom), including Gamma, Beta, Alpha, Theta, Delta rhythm.

This rhythmic conduction process of the heart is regulated by cardiac conduction system, which initiates and coordinates electrical impulses. This rhythm of heart starts from the sinoatrial node (SAN), the primary pacemaker located at the posterolateral junction of the superior vena cava and the right atrium, which spontaneously generates electrical excitation (Fig. 2a). The impulse propagates through the atria via internodal pathways, causing atrial contraction (synchronized right and left atrial depolarization, represented by the P wave on the ECG), and reaches the atrioventricular node (AVN) [24]. The AVN, located at the junction of the right atrium where the atria connect with the ventricles, introduces a delay (PR interval) between atrial and ventricular excitation, allowing atrial ejection before ventricular activation. Then the impulse travels through the bundle of His, bifurcates into the left and right bundle branches along the interventricular septum. Subsequently, the impulse spreads via Purkinje fibers on the inner surface of the endocardium to trigger contraction of ventricles (ventricular depolarization, manifested as the QRS complex). Finally, ventricular repolarization proceeds from epicardium to endocardium (corresponding to the T wave), which completes one action potential cycle.

The vector summation of the total electrical potentials generated by atrial and ventricular myocardial cells is captured as the ECG signals. A normal cardiac cycle of ECG waveform consists of sequential a P wave, a QRS complex, and a T wave (Fig. 2a inset and Table 1). It typically ranges from 10 μV to 4 mV in amplitude [20,27] with frequency components lies between 0.05 Hz and 150 Hz for adults [28] and the recommended minimum bandwidth for infants is between 0.05 and 250 Hz [29].

**Table 1:** Characteristics of ECG waveform components.

ECG waveform components	Physiological Meaning	Amplitude/Duration
P wave	Atrial depolarization	0.1–0.3 mV/<120 ms [25]
QRS complex	Right and left ventricles depolarization	Up to 3 mV/70–110 ms
T wave	Ventricular repolarization	∼300 ms (heart rate-dependent) [26]
PR interval	AVN delay	120–200 ms
QT interval	Ventricular action potential duration	320–440 ms

While resting ECG provides essential diagnostic information, dynamic ECG monitoring during physical activity or daily life is increasingly recognized for its unique clinical value. Dynamic ECG recording enables the detection of episodic arrhythmias, ischemic episodes, and conduction disorders that manifest only during movements [5,30,31]. For instance, exercise can unmask latent myocardial ischemia or arrhythmias that are absent at rest, thereby improving diagnostic sensitivity and supporting personalized rehabilitation guidance.

### 2.2 EMG

EMG records the electrical activity generated by muscle fibers and motor neurons during muscle contraction or tension. EMG shows great promise in clinical diagnostics, rehabilitation medicine, sports science and human-machine interface (HMI). It enables the diagnosis of neuromuscular disorders, assessment of muscle functional status, identification of movement intentions, tracking of rehabilitation progress, and optimization of athletic performance [32,33].

Muscles are structurally arranged by smallest functional units termed motor units (MUs), each consisting of a single motor neuron and the bundle of muscle fibers that the neuron innervates (Fig. 2b) [32]. The number of muscle fibers per MU varies significantly across muscle types, reflecting different functional demands. Generally, the fewer muscle fibers innervated by a neuron, the greater the precision of control over subtle muscle movements. Activation begins when the nervous system triggers a motor neuron, initiating an electrical impulse that travels along the axon to neuromuscular junctions—specialized synapses where motor neurons meet muscle fibers. There, neurotransmitter release induces depolarization of the muscle fiber membranes, initiating the sliding of myofilaments and ultimately leading to muscle contraction. The resulting electrical activity from a single MU is known as a motor unit action potential (MUAP), typically ranging from 20 μV to 2 mV in amplitude. The summation of action potentials from multiple MUs can be captured as surface EMG (sEMG) signal on skin.

A typical amplitude of the sEMG signals is 0.1 μV to 5 mV with dominant frequency components between 10 and 500 Hz [34]. The morphology and intensity of sEMG signals are governed by the number of simultaneous recruited MUs, their discharge frequency, as well as geometric relationship between muscle fibers and electrodes placement (*e.g.*, relative position, distance and orientation) [35]. Electrodes should be positioned over the muscle belly midpoint to maximize signal amplitude and SNR, while minimizing crosstalk from adjacent muscles.

SEMG is particularly susceptible to MAs owing to unavoidable, intrinsic muscle movements [36]. In detail, muscle contractions induce changes in muscle fibers length and cross-sectional area, causing subdermal tissue shifts that displace skin-mounted electrodes. It makes MA suppression a critical challenge even in a conventional sEMG recording scenario.

### 2.3 EOG

EOG captures variations in the corneal-retinal standing potential (CRP) caused by eye movements, serving as an effective method for monitoring ocular electrical activity. By tracking eye position and movement dynamics, EOG provides valuable insights into ocular behavior, supporting diverse applications, including ophthalmology (*e.g.*, strabismus, nystagmus, and retinopathy), sleep studies (*e.g.*, sleep disorders), human-computer interaction (*e.g.*, eye-writing systems), and psychophysiological monitoring (*e.g.*, emotion, fatigue, and stress assessment) [37].

An inherent electrical dipole exists within the eyeball, resulting from the negatively charged retina located at the back and the positively charged cornea at the front [38,39]. This dipole, typically 60-80 mV at rest, is aligned along the anteroposterior axis of the eye bulb (minimally deviating from the optical axis of the eye). When the eye moves, the dipole field reorients, inducing measurable potential shifts proportional to the gaze angle. It should be noted that EOG detects dipole displacement rather than the direct electrical activity of extraocular muscles.

EOG signals are characterized as non-stationary with a substantial DC component [40]. Their amplitude is typically ranging from 50 μV to 3.5 mV, with a frequency range extending from DC to 100 Hz [39,41]. The essential bandwidth lies 0.1-40 Hz [42]. Compared to EEG and EMG, EOG signals show higher amplitude and greater stability [43]. Moreover, a near-linear relationship exists between EOG signal amplitude and gaze angle within a specific angular range (±30° vertically and ±50° horizontally) [41], which typically yields 5-20 μV per degree. This linearity simplifies the interpretation and processing of EOG data. In terms of EOG recording methodology, a typical electrode configuration employs five electrodes (including one reference electrode) arranged around the eyes, which are positioned horizontally (right-left, R-L) and vertically (upper-lower, U-D) around the eyes to independently capture EOG signals for each channel (Fig. 2c). For instance, as the cornea moves toward a right-side electrode, the right-left potential difference increases; movement in the opposite direction reduces it.

Despite its utility, EOG recording remains susceptible to MAs arising from muscular activities (*e.g.*, blinking, facial movements) [40]. Although blinking does not alter the electrostatic potential of the eye, it can cause movement of the electrodes, leading to the emergence of MAs. Additionally, blinking may be mistaken for a saccadic movement due to their similar frequency and amplitude, both involving a reflexive vertical motion of the eyeball [40].

### 2.4 EEG

EEG originate from the postsynaptic potentials of pyramidal neurons within the cerebral cortex [44]. These signals reflect the comprehensive electrical activities across a large number of neurons, which propagate through biological tissues and are captured non-invasively by electrodes on the scalp. While invasive methods such as electrocorticography (ECoG) offer superior spatiotemporal resolution by recording directly from the cortical surface, EEG is still highly regarded for its non-invasive advantages. EEG records blended signals from multiple brain regions, enabling critical applications across clinical diagnostics (*e.g.*, of epilepsy and sleep disorder identification) [45,46], brain-computer interfaces (BCIs) that translate neural activity into control commands) [47], cognitive and behavioral research (*e.g.*, investigation of attention, memory and mental states) [48,49].

EEG signals exhibit lower amplitude (5–300 μV) and narrower bandwidth (0.5–100 Hz) compared to ECoG (0.01–5 mV; 0–200 Hz) [20]. EEG signals can be classified into two types: (1) Spontaneous EEG: continuous rhythmic activity generated by the cerebral cortex during rest or unstimulated states. (2) Evoked potentials (EPs): transient electrical responses elicited by specific sensory, cognitive, or motor stimuli, characterized by their waveform morphology and latency. Based on spectral characteristics, EEG signals can be categorized into different rhythms: Delta (0.1–4 Hz), Theta (4–8 Hz), Alpha (8–13 Hz), Beta (13–30 Hz) and Gamma rhythm (30–50 Hz) [50,51]. Each rhythm is associated with specific neurophysiological states. For more comprehensive coverage of EEG rhythms and their functional correlates, readers are directed to dedicated reviews [46,50].

In terms of EEG recording methodology, the international standardized 10-20 system with precisely positioned 19 electrodes (Fig. 2d) [52], are essential for consistent spatial mapping of neural activity. Each electrode is designated according to the regions of Fp (frontal polar), F (frontal), C (central), P (parietal), T (temporal), O (occipital), with odd and even numbers representing the left and right hemispheres, respectively. The designation “z” indicates the midline [53]. Reference electrodes are typically placed at the nasion, inion, and auricular sites (A1/A2) to further enhance measurement consistency.

MAs significantly impact EEG signal quality, especially in ambulatory or real-world settings. These artifacts arise from physical movements such as head motion, facial muscle activity, or electrode displacement, introducing high-amplitude, non-neural noise into the recorded signals. Generally, such MAs can obscure genuine neural activity, complicating data interpretation in applications like BCIs or seizure detection.

Table 2 shows the typical amplitude and frequency ranges of primary EP signals, which are prevalently weak with frequency distributions mainly located at the low frequency [54]. Thus, it is desirable to maximize the amplitude of the targeted EP signals while minimizing MAs.

**Table 2:** Characteristics of representative electrophysiological signals.

EP signal	Magnitude	Dominant frequency range [Hz]
ECG	0.01−4 mV	0.05−150
sEMG	0.1−5 mV	10−500
EOG	0.05−3.5 mV	0.1−40
EEG	5−300 μV	0.5−100

## 3 Sources of bioelectric motion artifacts and skin-electrode interface

To effectively suppress MAs at the source, a thorough understanding of the underlying generation mechanisms is essential. MAs emerge from a complex interplay of mechanical and electrical phenomena at the skin–electrode interface. Although the complete picture of MA formation remains under investigation, it is generally accepted that MAs originate from relative motion between the electrode and the skin [55], which leads to dynamic instabilities at the skin–electrode interface [56]. External mechanical stresses (*e.g.*, lateral shear, vertical and torsional forces) may induce relative motion, slippage and deformation at the interface, especially for dry electrodes [5]. These mechanical stresses alter key electrical properties of both the electrode and the interfacial zone [57,58]. Even subtle motions, such as respiration, can change the interfacial impedance and skin potential [59]. Furthermore, under prolonged use, cyclic mechanical loading accumulates stress, resulting in electrode detachment or fatigue failure, which exacerbates the generation of MAs over time. Therefore, maintaining a stable and robust skin–electrode interface during movement is crucial for high-quality, motion-artifact-free EP signal acquisition.

Based on the electromechanical origins, MA generation mechanisms can be categorized into three primary types: impedance instability, biopotential instability, and friction. Impedance instability arises from changes in the contact impedance between the electrode and skin. Biopotential instability is caused by biopotential changes including the distribution of electrolytes, half-cell potential, and the structure of the double electric layer. Triboelectric effects also contribute to charge accumulation at the interface.

### 3.1 Interfacial impedance instability

A principal source of MAs lies in the dynamic fluctuation of impedance at the electrode–skin interface [60,61]. Changes in effective electrode–skin contact area, interfacial pressure, interfacial air gaps, skin hydration (*e.g.*, sweating), perspiration accumulation at the interface or stratum corneum condition can all contribute to this impedance instability. Such variations directly influence captured signal amplitude and quality, since the electrode–skin impedance forms part of a voltage divider network with the input impedance of the front-end amplifier. Generally, a low electrode–skin impedance leads to a larger EP signal amplitude, while an increase in impedance leads to attenuation of the signal and the decrease of SNR [11,62].

**Fig. 3:**
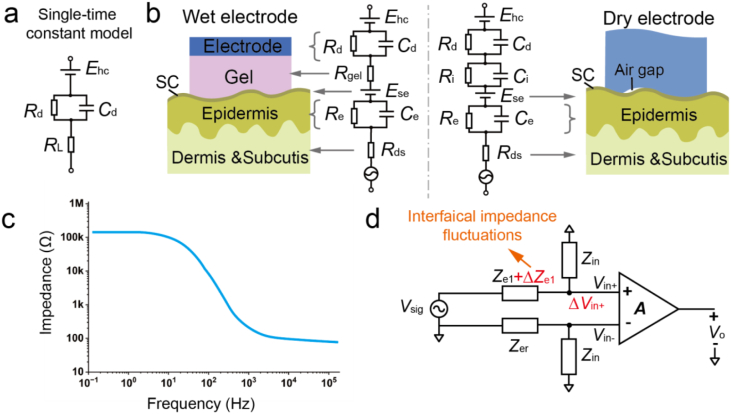
Equivalent circuit model and impedance spectrum of skin-electrode interface impedance. (a) Single-time constant model of the skin-electrode interface impedance. (b) Multi-time constant models of wet and dry electrode. (c) Frequency-dependent interfacial impedance spectrum. (d) Generation of MAs due to changes in interfacial contact impedance of the capturing electrode after voltage division through the front-end amplifier.

The electrode–skin impedance characteristics are commonly described using time constant equivalent circuit models that incorporates both a series of parallel or series RC components (Fig. 3a) [63]. While a simple single-time constant model (Swanson-Webster model) can offer a basic representation for all types of electrodes (Fig. 3a), it does not effectively account for the multi-layered complexity of skin tissue. More accurate models utilize double or multiple time constants to represent different skin layers and electrode types. For instance, wet electrodes and dry electrodes exhibit distinct impedance behaviors due to differences in ionic pathways and contact mechanisms (Fig. 3b). In a typical multi-time constant model (Fig. 3b), the impedance can be expressed as follows:

(3.1) $$Z_{\text{wet}}=\frac{R_{d}}{1+j\omega R_{d}C_{d}}+R_{\text{gel}}+\frac{R_{e}}{1+j\omega R_{e}C_{e}}+R_{\text{ds}}$$

(3.2) $$Z_{\text{dry}}=\frac{R_{d}}{1+j\omega R_{d}C_{d}}+\frac{R_{i}}{1+j\omega R_{i}C_{i}}+\frac{R_{e}}{1+j\omega R_{e}C_{e}}+R_{\text{ds}}$$

In this model, ω is the angular frequency. Circuit elements represent the resistance (*R*_e_) and capacitance (*C*_e_) of the epidermis layer, the combined resistance (*R*_ds_) of the dermis and subcutis layers, the double-layer capacitance (*C*_d_) and charge transfer resistance (*R*_d_) at the electrode–skin interface, along with the half-cell potential of between metal and electrolyte (*E*_hc_), and half-cell potential of semi-permeable stratum corneum (*E*_se_) due to metabolic difference between the dead and living epidermal cells on either side of the stratum corneum [64]. Increasing the contact area between the electrode surface and the skin can increase the value of *C*_d_, while the value of *R*_d_ would be decreased. A critical difference between wet and dry electrodes is that wet electrodes include an electrolyte resistance (*R*_*gel*_), while dry electrodes feature an interfacial layer (*R*_i_ and *C*_i_) due to poor contact. *R*_i_ is typically much larger than *R*_gel_. Moreover, indirect effects (such as modulus mismatch, poor contact and inconsistent adhesion), lead to interfacial separation and void/gap formation [65], which further disrupt overall interface impedance under motions.

The presence of perspiration further complicates the impedance profile. Perspiration, which is rich in ions, can create conductive pathways across the skin and enhance the hydration of the skin, reducing the overall impedance and introducing variable ionic sources. These dynamic changes are difficult to quantify and are often not depicted in equivalent circuit models.

The electrode–skin impedance is highly frequency-dependent (Fig. 3c), predominantly due to capacitive components. At low frequencies within the typical bandwidth of most EP signals, the impedance is dominated by resistive elements and remains high. As frequency increases, capacitive pathways progressively short-circuit, causing a sharp drop in impedance magnitude. This frequency-dependent impedance can be characterized using electrical impedance spectroscopy (EIS), enabling extraction of each component values in the equivalent model.

To describe the non-ideal capacitive behavior more accurately, constant phase elements (CPEs) are commonly utilized in place of ideal capacitance [[66], [67], [68]]. The impedance of a CPE can be expressed as follows:

(3.3) $$Z_{CPE}=\frac{A}{{\left(j\omega \right)}^{n}}$$

Where *A* is a constant that reflects the pseudocapacitive impedance characteristics of the CPE [68]. The parameter *n* is a dimensionless quantity (0 ≤ *n* ≤ 1) that is closely associated with interfacial heterogeneity. Both parameters *A* and *n* are independent of frequency. When *n* = 1, *Z*_CPE_ corresponds to an ideal capacitance (phase angle of −90°), whereas when *n* = 0, *Z*_CPE_ ​ represents a pure resistive behavior. When 0< *n* < 1, it arises from continuously distributed time constants for charge-transfer reactions and surface heterogeneity [69], such as non-uniform sweat gland distribution, contact imperfections, inhomogeneity of skin and nonuniformity of electrode surfaces [70]. For ordinary skin, the value of n is generally taken as 0.8 [69].

To illustrate the impact of impedance fluctuations, consider a simplified biopotential measurement setup with a pair of electrodes (recording and reference electrode) is connected severally to the input of the front-end biopotential amplifier (Fig. 3d). The actual input voltage *V*_in +_ seen by the amplifier depends on the voltage division between the skin–electrode impedance (*Z*_e1_, *Z*_er)_ and the amplifier's input impedance (*Z*_in_, considered as a pure resistor for simplicity) [71]. If *Z*_e1_ and *Z*_er_ are constant, *V*_in +_ can be expressed as:

(3.4) $$V_{in+}=V_{\text{sig}}\frac{Z_{\text{in}}}{Z_{e1}+Z_{\text{in}}}$$

Where *V*_sig_ represents EP signals. If skin-electrode impedance fluctuates by ΔZ_e1_ due to human motions, this generates an MA voltage Δ*V*in ^+^ proportional to ΔZ_e1_.

(3.5) $$\Delta V_{in+}=V_{\text{sig}}\frac{Z_{\text{in}}}{\Delta Z_{e1}+Z_{\text{in}}}$$

If ΔZ_e1_ becomes comparable to Z_in_, the resulting MAs can significantly corrupt the measured EP signal. Similarly, the fluctuations in impedance *Z*_er_ of the reference electrode can also generate MAs. Thus, the input impedance of amplifier is required to significantly exceed the skin-electrode impedance (typically Z_in_ ≥ 1 GΩ) to reduce the impact of ΔZ_e1_, minimizing EP signal attenuation and MA amplitude under motions [62]. Besides, decreasing the skin-electrode impedance is also a way to minimize EP signal attenuation and MA amplitude, particularly important for dry electrodes (impedance fluctuations can reach 100-1000 Ω) that commonly exhibit large impedance than wet electrodes counterparts.

Generally, the stability of skin-electrode impedance can be improved by conformal electrode materials, skin pretreatment (*e.g.*, exfoliation or skin abrasion), and optimized electrode fixation.

### 3.2 Biopotential instability

In addition to impedance instability, MAs can originate from biopotential instability at the skin-electrode interface. Skin deformation (*e.g.*, stretching, compression, or rotation) caused by motions, alters the endogenous potential difference (∼30 mV) across the epidermis, known as the injury current, which is generated by metabolic differences between the inner living skin layers and the outer dead stratum corneum [24,72]. The deformation modifies the current pathways that injury current flows through, direct changing the epidermis potential (up to several millivolts in magnitude) [73,74], which is the dominant contributor to this type of MAs. It should be noted that the potential changes do not appear to result from alterations in skin impedance [73].

Besides, a unique interface forms between human skin (regarded as an electrolyte layer) and the electrode (regarded as an electric conductor), known as the electrode-electrolyte interface. At this interface, the exchange of ions and electrons occurs. To balance charge, ions in the electrolyte rearrange near the electrode surface, forming two charged layers: one tightly adhered to the electrode surface (the inner Helmholtz layer), and one diffusing into the electrolyte layer (the diffuse layer). This structure is known as the electrical double layer (EDL). When the electrode undergoes relative sliding with the skin or when the contact pressure/area experiences significant fluctuations, it will disrupt the double electric layer at both the physical and chemical levels, which are the sources of MAs. In physical level, sliding directly shear the EDL, causing instantaneous disordering of charge distribution and generating large transient potential differences. Additionally, since the capacitance of the EDL is proportional to the contact area, fluctuations in area lead to dramatic changes in interfacial capacitance, resulting in signal baseline drift. In chemical level, the stable ionic environment formed by trace sweat (a natural electrolyte) on the skin surface is compromised. Sliding may dry out the local area or introduce uneven sweat distribution, disrupting the electrolyte layer. Furthermore, sliding causes ions beneath the electrode to be either “squeezed away” or “accumulated,” altering local ion concentration and directly modifying the potential of the EDL.

The electrode type strongly influences the susceptibility to such biopotential instability. Dry electrodes, which often rely on a thin and inconsistent moisture layer for electrical contact, are particularly sensitive to movements. Even slight movements can compress or deform the sweat/electrolyte layer, leading to significant changes in the ionic concentration gradient within this layer [75]. This disrupts the electrochemical equilibrium at the electrode-electrolyte interface, leading to notable fluctuations in the half-cell potential [63]. In contrast, gel-based electrodes exhibit greater stability owing to the buffering effect of the hydrogel layer, though motion can still perturb the electrical double layer at the metal–gel interface, generating artifact voltages within the electrochemical cell [76].

It is also noteworthy that the transepidermal potential also exhibits slow drifts unrelated to skin deformation, influenced by sympathetic nervous activity. Emotional states (*e.g.*, fear, excitement and happiness) can induce millivolt-level fluctuations through sympathetic sweating and modulated transcutaneous water loss [77].

Abrasion or puncture of the stratum corneum can reduce MAs by eliminating the contribution of the epidermal potential source (*E*_se_) and modifying *R*_e_ and *C*_e_. However, this approach requires skill to perform with risks of skin irritation as well as infection, and offers only temporary benefit since the stratum corneum regenerates within approximately 24 h [78,79].

### 3.3 Friction

Friction at the skin-electrode interface during movements can generate MAs through triboelectric effects [13,15,80]. When two materials (such as electrode substrate and skin), contact, separation, or slide against each other, charge transfer occurs, resulting in charge accumulation on the electrode. In addition, these surface charge on charged materials generally diminishes with time [80]. This phenomenon introduces broadband artifacts that is particularly challenging to filter. Poor contact (*e.g.*, gaps and voids) between the skin and electrode exacerbates this issue. Intermittent contact or microscopic separation under motion creates rapid charge separation and recombination events, generating high-frequency noise components.

This effect can be modeled as a current source dQ(*t*)/d*t*, where Q(*t*) represents the time-varying residual charge on the electrode [80]. However, there are still no satisfying quantitative models exist to generally predict MA magnitude under various materials and motion conditions.

Minimizing friction is critical, not only for signal integrity but also for wearer comfort and the prevention of skin irritation. Thus, strategies include improving interfacial conformability, using low-friction materials, and minimizing relative sliding.

## 4 Source-level motion artifacts suppression strategies

To effectively suppress MAs at their source, various source-level approaches that focusing on innovative electrode designs have been proposed. Rather than dealing with signals contaminated by MAs after acquisition, these approaches proactively stabilize the skin-electrode interface against mechanical disturbances. Generally, source-level MAs suppression strategies can mainly be classified into four categories, including the skin-anchoring strategy, deformation constraint strategy, stain-insensitive strategy and damping strategy. These strategies aim to prevent artifact generation by addressing the fundamental mechanisms discussed earlier—impedance instability, biopotential fluctuations, and friction. The following sections systematically analyze these strategies, highlighting their underlying principles, representative implementations, and comparative performance.

### 4.1 Skin-anchoring strategy

The skin-anchoring strategy minimizes MAs by strengthening electrode-skin anchoring, which is mainly targeted at solving interfacial impedance instability and friction. This strategy reduces the relative slip, micro-delamination and air gap formation at the electrode-skin interface by improving interfacial adhesion, conformality, friction or contact pressure, thereby stabilizing the electrical contact between electrode and skin, eliminating the dynamic fluctuation of interfacial impedance and the triboelectric charge accumulation caused by friction [56,81]. Ultimately, the strategy achieves MA suppression by ensuring the electrode moves synchronously with the skin during body movements, and the quantitative suppression effect is reflected in the significant improvement of SNR and the maintenance of stable baseline.

**Fig. 4:**
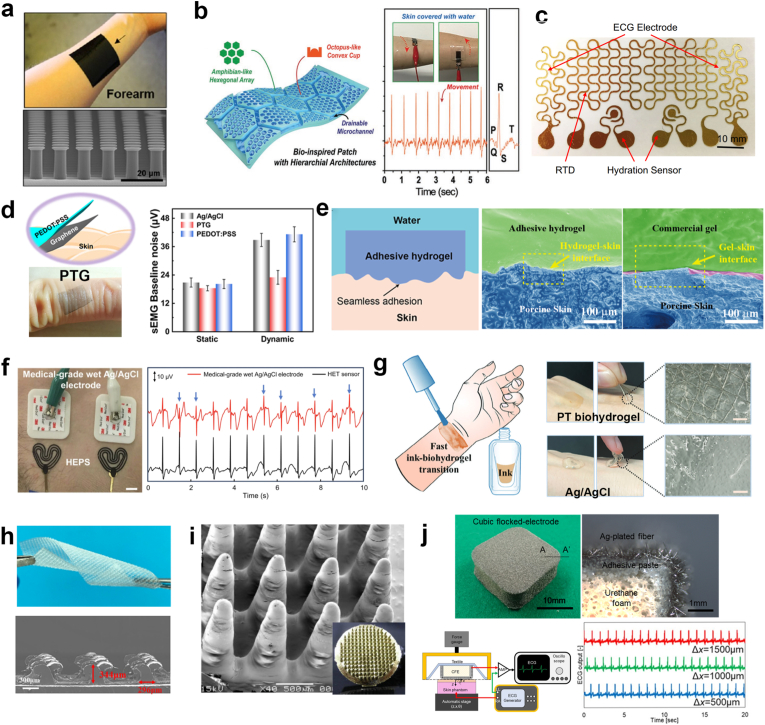
Skin-anchoring strategy. (a) Photo and SEM image of the conductive dry adhesive electrode with gecko-inspired micropatterns. Reproduced with permission [82]. Copyright 2016, American Chemical Society. (b) Schematic illustration of patch with the amphibian and octopus-like hierarchical architectures (left). ECG signal measurement on wet skin in dynamic movements by the patch (right). Reproduced with permission [83]. Copyright 2019, Wiley-VCH. (c) Top view of the e-tattoo. Reproduced with permission [84]. Copyright 2018, Springer Nature. (d) Schematic illustration and photograph of PTG conformed on a finger (left). sEMG baseline noise comparison of the three types of electrodes in both static and dynamic status (right). Reproduced with permission [36]. Copyright 2021, Springer Nature. (e) Illustration of the proposed adhesive hydrogel (left). The commercial electrode with voids existing at the interface cause water permeation and adhesion deterioration, while the proposed adhesive hydrogel can seamless adhesive to the skin for stable EP signals recording (right). Reproduced with permission [85]. Copyright 2023, American Chemical Society. (f) The ECG signals recorded simultaneously by both HEPS and medical-grade wet Ag/AgCl electrodes demonstrated a significantly lower susceptibility of the HET sensor to motion (scale bar: 1 cm). Reproduced with permission [86]. Copyright 2024, Springer Nature. (g) The schematic of the on-skin paintable PT biohydrogel (left). Ultradepth-of-field microscopy images of the PT biohydrogel film containing the skin's surface topography after removing, indicating conformal contact with the skin, while it is absent in the gel of commercial ECG electrodes (right). Reproduced with permission [87]. Copyright 2024, Wiley-VCH. (h) Photograph and SEM image of the bioelectrode with Fructus xanthii-inspired barbed structure. Reproduced with permission [88]. Copyright 2022, American Chemical Society. (i) SEM image and photo of the 3D printed electrode of conical needles array. Reproduced with permission [89]. Copyright 2012, Elsevier. (j) Photograph of the CFE along with a cross-sectional view at the A-A′ line (top). MA evaluation experiments conducted during x-axis movement of the automatic stage (bottom). Reproduced with permission [59]. Copyright 2022, Springer Nature.

One method to improve the electrode's anchoring to the skin is to boost the electrode's adhesion to the skin. The enhanced adhesion can minimize motion artifacts and reduce the probability of micro-delamination and micro-motion at the electrode interface [8]. By mimicking the microstructures in nature (*e.g.*, gecko toes micropillars, octopus suction cups), these bio-inspired designs function as multiple skin-anchoring points, effectively reducing the relative slippage between the electrode and the skin. Fig. 4a shows high aspect ratio, elastic carbon nanocomposite electrodes with high dry adhesion, fabricated by mimicking the microstructure of gecko toes with micropillar arrays [82]. The dry adhesion of the micropillar structure significantly enhance the electrode-skin interfacial adhesion, which effectively eliminates the relative slip between electrode and skin under extreme exercise. Inspired by the microchannel network in the toe pads of tree frogs and convex cups in the suckers of octopi, a hierarchical architectures patch with octopus-like convex cups in the hexagonal structures and water drainable microchannels is reported (Fig. 4b), showing improvement in skin adhesion in dry, sweaty and flowing water conditions [83]. By employing an ultrathin layer of reduced graphene oxide (rGO) nanoplatelets on top of these microstructures, ECG signals were monitored using this patch without delamination from dry/wet skin under hand movements. However, it should be pointed out that these fine microstructures typically involve complicated and costly manufacturing processes (*e.g.*, photolithography). In addition, their adhesion to the skin is easily compromised by secreted sweat, skin contaminants, and the microstructural damage, which can also induce discomfort through suction-based mechanisms. Thus, electrode materials inherently possess exceptional adhesive properties are also reported to reduce MAs [8,56,[90], [91], [92]]. An ultrathin polymeric conductive adhesive epidermal electrode by blending PEDOT:PSS with polyvinyl alcohol (PVA) and d-sorbitol, which significantly enhances adhesion through abundant hydrogen bonding [8]. The resulting high adhesion and low elastic modulus enable the electrode to maintain conformal contact with the skin during significant and repetitive deformation, exhibiting minimal baseline fluctuations and achieving high SNR (28.15 dB in case of running) in EMG monitoring as well as long-term ECG recording for one week even under repetitive and diverse movements. Another example is an intrinsically adhesive and conductive hydrogel, which incorporates catechol groups to achieve robust adhesion, which effectively reduces motion artifacts during dynamic recordings [91]. Consequently, the hydrogel enabled high-quality biopotential monitoring, achieving stable ECG, EMG signals with minimized interference during various body movements and successfully recording epileptic ECoG signals in freely moving rats.

Improving electrode conformality to skin is a widely utilized approach to enhance skin-anchoring [93]. Conformality (conformal contact) refers to the intimate and seamless contact of electrodes to the non-planar skin topography and follows the displacement of skin exactly [11], eliminating air gaps at the electrode-skin interface. The conformal contact increases the effective contact area, reduces interfacial impedance, facilitates signal transfer across the interface, and allows synchronous movement between electrode and skin with reduced relative motion [11,31,58,84]. The pursuit of conformality has driven innovations in skin-modulus-matched electrodes. Modulus matching is commonly a prerequisite for achieving conformal contact. The conformal contact can be determined by considering the total energy of the interface (*U*_interface_), which includes the bending energy of the electrode (*U*_bending_), the elastic energy of the skin (*U*_*skin_*elasticity_), and the adhesion energy of contact (*U*_adhesion_) [11].

(4.1) $$U_{\text{interface}}=U_{\text{bending}}+U_{\text{skin}\_\text{elasticity}}+U_{\text{adhesion}}$$

The bending energy depends on the effective bending stiffness of the electrode composite, which is positively correlated with the effective moduli of its constituent materials and, most significantly, strongly positively correlated with the thicknesses of constituent layers. The elastic energy of the skin is mainly determined by the wavelength and modulus of the skin. Conformal contact is achieved when the adhesion energy *U*_adhesion_ outweighs the sum of the bending and elastic energies (*U*_bending_ + *U*_*skin_*elasticity_). Moreover, the electrical contact impedance (*Z*_C_) at the interface is directly influenced by the quality of this contact. It can be modeled as:

(4.2) $$\left|Z_{C}\right|\approx \frac{\rho d}{A\sqrt{1+{\left(\omega \rho \varepsilon \right)}^{2}}}$$

where *ρ* is skin resistivity, *d* is inter-electrode distance, *A* is the effective contact area, ω is signal frequency, and *ε* is skin dielectric constant. Fluctuations in the effective contact area lead to fluctuations in the interface impedance. Thus, modulus matching reduces the interfacial impedance and the motion-induced impedance fluctuations, which can effectively suppress fluctuations within a range of tens to hundreds of ohms, even without the utilization of conductive gel [81]. In contrast, electrodes with a significant modulus mismatch may experience impedance fluctuations on the order of kilo-ohms. This represents an approximate order-of-magnitude reduction in impedance variation, which fundamentally minimizes MAs by stabilizing the electrode-skin interface.

Thickness reduction is one approach to match the modulus with skin. As the substrate thickness decreases, the effective modulus is significantly reduced, thereby enhancing flexibility and mechanical compatibility with skin. Electronic tattoos (e-tattoos) exemplify in conformal design, with thicknesses ranging from nanometers to micrometers enabling van der Waals adhesion without additional adhesives [11,94]. These devices can follow skin deformation without mechanical constraint, effectively moving as a second skin layer. For example, a low-cost open-mesh e-tattoo fabricated through modified cut-and-paste methods achieve conformal attachment through their extreme thinness (1.5 μm) (Fig. 4c) [84], demonstrating high-fidelity sensing with minimal MAs under various body movements. Similarly, ultra-thin (∼100 nm) PEDOT:PSS/CVD-graphene dry electrodes (PTG) combine ultra-conformability with mechano-electrical stability (Fig. 4d) [36]. The ultra-thin configuration offers superior conformal contact with skin, eliminating interfacial air gaps and ensuring synchronous movement with the skin. The PTG can accurately monitor ECG, sEMG, and EEG with lower baseline noise than Ag/AgCl gel electrodes. However, these free-standing e-tattoos face challenges regarding robustness and reusability due to their inherent fragility [95].

Beyond thickness reduction, electrodes with skin-like mechanical properties (tens of kPa to 1 MPa in modulus) can conformally contact with the wrinkled skin, following skin deformation [15]. Hydrogels have emerged as ideal candidates for this purpose, combining tissue-like modulus, softness, and biocompatibility [[96], [97], [98], [99], [100]]. Recent advances include an ultra-low modulus (4.5 kPa) poly(acrylic acid)/chitosan/MXene-based conductive hydrogel electrode that fabricated without chemical crosslinker agents [85]. This electrode exhibits stable and seamless adhesion in both air and aquatic conditions without adhesion deterioration, effectively preventing water permeation into the interfacial void that observed in conventional gel electrodes (Fig. 4e). Similarly, an ultra-conformal, breathable epidermal electrode with tissue-like low modulus (0.53 MPa) and strong adhesion was developed [101]. The electrode maintains dynamically stable skin contact and low contact impedance during movements and even heavy sweating, thereby enabling long-term, high-fidelity monitoring of EP signals (ECG, EMG, EOG, EEG) in complex environments like exercise and even heavy-sweat conditions. However, traditional hydrogel electrodes are commonly constrained by inherent limitations, most notably dehydration-induced mechanical stiffening over time, which causes increased interfacial impedance and compromises conformality with the skin [102]. Thus, a low thickness (20 μm), ultra-softness (31 kPa at 37 °C) stretchable gelatin-ployacylamide-polypyrrole hydrogel-based e-tattoo (HET) was proposed with exceptional water retention capabilities (maintaining performance over 6 months) (Fig. 4f) [86]. The outstanding water retention capability is achieved through a strategic combination of a parylene C barrier layer (200 nm thick) and the incorporation of water-retention agents, which synergistically minimize dehydration from the hydrogel matrix. In comparison to the gold standard Ag/AgCl electrodes, HET-based electrophysiological sensors (HEPS) show reduced motion susceptibility and a significant enhanced SNR (up to 19 dB). This superior performance is attributable to the exceptional skin conformability, which results in an electrode-skin impedance that is 234% lower than that of Ag/AgCl electrodes.

Paintable hydrogel-based electrodes can also achieve conformal adhesion to the skin through on-skin rapid gelation of liquid precursors [87,[103], [104], [105]]. They adapt to skin wrinkles and curvature prior to curing, forming stable and customized electrode-skin interface. As shown in Fig. 4g, polyvinyl alcohol (PVA)-tannic acid (TA) biohydrogel (PT biohydrogel) ink undergo iquid-gel transition within 2 min through solvent evaporation [87], establishing intermolecular cross-linking between PVA and TA. Good dynamic compliance and conformability after gelation of the biohydrogel-based bioelectrode creates compliant interfaces resistant to MAs during body movements. Beyond their conformal properties, the electrode offers water-resistant adhesion and long-term stability, supporting high-quality electrophysiological monitoring over extended periods.

Friction enhancement at the interface and contact pressure improvement offer alternative pathways to reduce the relative slippage under motions. Bioinspired structural designs, such as the Fructus xanthii-mimetic barbed structure integrated into Ag/AgCl-thermoplastic polyurethane (TPU) electrodes, significantly enhances dynamic friction at the electrode-skin interface (Fig. 4h). This approach reduces interfacial sliding effectively during motion [88]. In addition, increasing contact pressure between the electrode and skin through non-penetrating topological features also enhances mechanical anchoring. Inspired by a pneumatic actuator, a fabric-based, shape-morphing bioelectrode (FLEXER) is integrated into clothing [106]. FLEXER uses air pressure to transform from a flat state into a 3D “scissor-jack” structure, actively pressing its MXene-based conductive center electrode against the skin for EP signal sensing. The design reduces motion artifacts by providing a stable, conformal, and evenly distributed contact force through its multi-legged symmetric structure. Unlike simple balloon-type contacts, the more uniform contact force reduces MAs caused by poor or uneven contact, minimizing fluctuations in skin-electrode contact during movement. Besides, 3D-printed dry electrodes with conical needle arrays (Fig. 4i) concentrate contact pressure at discrete points without sub-dermal insertion [89]. Another example is a cubic flocked electrodes (CFE) comprising urethane foam electrostatically flocked with Ag-plated fibers (Fig. 4j) [59]. This electrode utilizes shear-deformable structures to maintain adequate contact pressure (>500 Pa), effectively suppressing MAs induced by respiration and multi-axis movements. However, a critical trade-off exists between interfacial stability and biocompatibility. Excessive friction, pressure or rigid microstructures may cause skin irritation, erythema, or discomfort during prolonged use. Further advancements may focus on dynamically adaptive interfaces that maintain optimal contact across varying physiological conditions.

**Core Material and Structural Design Commonalities of Skin-anchoring Strategy**: The skin-anchoring strategy is based on the core design principle of strengthening the anchoring of the electrodes to skin. The representative materials/structures share the following key features:

i) Core material properties

**Skin-matched low modulus**: The elastic modulus of materials is in the range of kPa to low MPa (consistent with human skin modulus), avoiding mechanical mismatch-induced interfacial separation.

**High interfacial adhesion/conformability**: Dry/wet adhesion, or ultrathin-induced van der Waals conformal contact.

ii) Structural design commonalities

**Bio-inspired microstructures**: Gecko-like micropillars, octopus-like convex cups, Fructus xanthii-inspired barbs for multi-point anchoring.

**Ultra-thinning**: Nanometer/micrometer thickness (e-tattoos) to enhance conformability.

The Balance between interfacial stability (high adhesion/friction/pressure) and biocompatibility/comfort/easy removal, while avoiding skin irritation caused by excessive pressure or rigid microstructures during long-term wear, is necessary. Thus, it is necessary to make some trade-offs when designing.

### 4.2 Deformation constraint strategy

The deformation constraint strategy is mainly targeted at solving interfacial impedance and biopotential instability. Unlike the skin-anchoring strategy that follows skin movement, this strategy mechanically isolates the electrode sensing area from the surrounding skin deformation by adding strain-isolating structural elements, which limits or redirects the mechanical stress/strain on the skin surface, prevents the sensing area from being affected by epidermal deformation, and thus stabilizes the epidermal potential and interfacial impedance. This strategy is particularly effective for MAs generated by large-area skin deformation (*e.g.*, chest stretching), and its quantitative effect is typically reflected in the low strain (≤3%) of the electrode sensing area under physical activities.

**Fig. 5:**
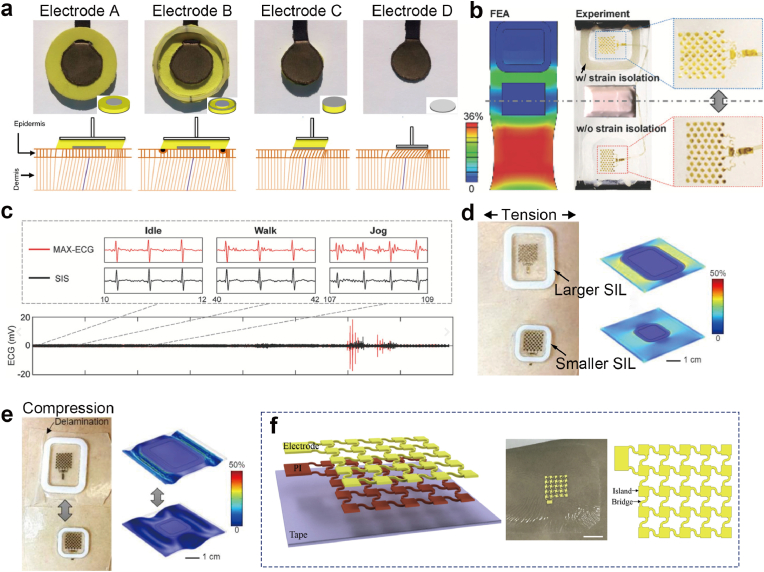
Deformation constraint strategy (a) Images and conceptual models of the different electrodes, along with an illustration of how the electrode structure affects the skin layers. Reproduced with permission. [72]. Copyright 2015, BioMed Central. (b) Comparison of FEA (left) and experimental result (right). The strain isolator (top) maintains sensor shape on the skin, while the electrode without the strain isolator experiences strain. (c) Comparison of raw ECG data throughout a full exercise session from both a commercial device (MAX-ECG) and the proposed system, including zoom-in segments during idle, walking, and jogging. Reproduced with permission [5]. Copyright 2021, Wiley-VCH. Validation of strain isolation via a comparison between large and small SIL under both (d) tension and (e) compression on the chest. Reproduced with permission [58]. Copyright 2022, American Chemical Society. (f) Schematic diagram and image of tape-based three-layer dry electrode and its island-bridge structure. Reproduced with permission [107]. Copyright 2020, Elsevier.

Structural isolation designs through padding, rings and ridges strain-isolating structures have demonstrated remarkable effectiveness in stabilizing the sensing interface and managing mechanical force distribution. For instance, textile electrodes with extended support structure beyond the electrical contact area can stabilize the skin-electrode interface by distributing mechanical stress over a wider skin area and minimizing localized skin deformation [72]. As shown in Fig. 5a, Electrode A (featuring a soft padding extending beyond the electrical contact area) and Electrode B (incorporating a skin deformation restricting ring outside the electrical contact area) demonstrated superior MA suppression compared to electrodes without padding (Electrode C) or rigid supports (Electrode D). The incorporation of the restricting ring further stabilizes the epidermis by transferring movement forces deeper into the dermis—a layer less prone to generating artifact-inducing potentials compared to the epidermis. This structure design addresses interfacial impedance variations and surface potential changes.

Another advanced implementation includes a fully-integrated wearable bioelectronic system with specialized strain isolators (SIL) surrounding a pair of open-mesh electrodes (SIS) (Fig. 5b) [5]. These strain isolators shield the electrodes from external strain and vibration transmission while maintaining conformal contact with the skin. Under a 15% tensile strain, the electrode without SIL protection experienced 36% strain, whereas the electrode shielded by the SIL exhibited only 3% strain. In simultaneous comparative tests with commercial wireless ECG monitors, the SNR of the commercial devices decreased by 53% during the transition from rest to jogging, while the SIS showed a reduction of only 16% (Fig. 5c). Similarly, an optimized strain-isolation layer biopatch design involves reducing the SIL footprint to mitigate strain concentration on surrounding skin of electrodes (*e.g.*, stretching, compression) and minimize delamination risks (Fig. 5d and e) [58]. By confining mechanical deformation to areas outside the SIL while simultaneously maintaining essential conformal contact with the skin, the electrodes can be protected from buckling/sliding, maintaining stable skin-electrode contact impedance. Finite element analysis (FEA) and human trials show that SIL restricts electrode strain to below 2% during physical activities while simultaneously lowering strain accumulation in surrounding elastomer and skin. Moreover, a compact SIL outperforms the loose SIL in performance by reducing deformation and preventing delamination from the skin under tension/compression. By balancing mechanical isolation with conformal skin contact, the optimized SIL design effectively suppresses MAs without compromising signal acquisition stability.

Island-bridge structures represent another approach to deformation constraint [108], particularly valuable for applications requiring both MA suppression and mechanical stretchability. This approach confines the sensing function to stable rigid “islands” while accommodating deformation through stretchable “bridge” interconnects. The rigid islands maintain stable electrical properties by isolating electrodes from mechanical stress, while the bridges enable stretchability without compromising electrical continuity [109,110]. For instance, a flexible and stretchable electrode array with island-bridge structures is fabricated, where the electrode islands are interconnected by serpentine-shaped bridges (Fig. 5f) [107]. The multilayer design of the electrode array consists of a stretchable tape substrate, a polyimide (PI) intermediate layer, and a patterned Au electrode layer. This structure allows electrodes to maintain conformal contact while accommodating significant skin deformation (up to 10% strain), with demonstrated effectiveness in preserving ECG signal features during running tests.

Despite their effectiveness, deformation constraint strategy faces certain limitations. The isolation structures typically reserve only the central region for bioelectrical acquisition, potentially reducing electrode array density compared to unrestricted designs. This trade-off may limit spatial resolution in mapping applications. Additionally, isolation structures may cause discomfort or restrict natural movement in some applications, particularly during high-intensity activities. Nevertheless, for scenarios where signal stability outweighs these considerations, deformation constraint offers effective MA suppression.

**Core Material and Structural Design Commonalities of Deformation Constraint Strategy**: The deformation constraint strategy is based on the core design principle of isolating sensing area and redirecting mechanical strain. The representative materials/structures share the following key features:

i) Core material properties

**High strain isolation capability**: The strain-isolating/island materials commonly have high Young's modulus (∼GPa), which can effectively block or buffer the transmission of skin surface strain/stress.

ii) Structural design commonalities

**Strain-isolating structures**: Padding, restricting rings, or independent strain isolators surrounding the sensing area.

**Island-bridge structure**: Rigid islands serve as the sensing areas, isolating them from mechanical strain, while the stretchable bridges act as interconnects to ensure electrical continuity.

### 4.3 Strain-insensitive strategy

The strain-insensitive strategy is mainly targeted at solving interfacial impedance instability that caused by dynamic changes in electrode internal resistance. Mechanical deformation (stretching/compression/twisting) of the electrode can cause the elongation and breakage of internal conductive pathways as well as the dynamic change of electrical resistance, which thus directly introduces MAs into EP signals [[111], [112], [113], [114]]. This strategy achieves MA suppression by designing deformation-insensitive conductive materials or strain-localized structural designs, which maintain the stability of electrode internal resistance (small resistance change rate) and interfacial impedance under large electrode deformation.

**Fig. 6:**
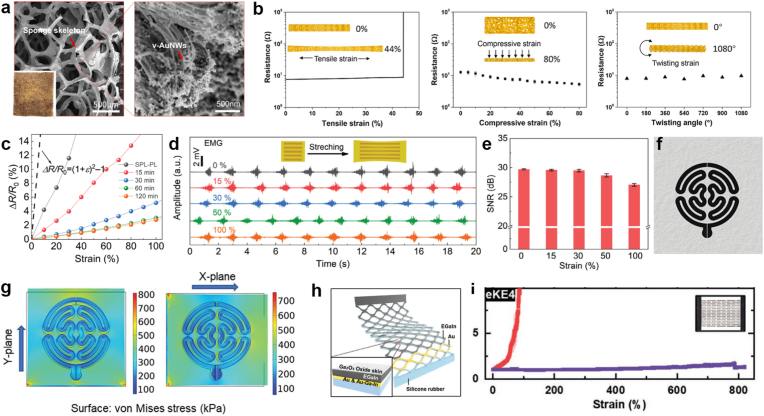
Stain-insensitive strategy. (a) Photograph and SEM images of the G-foam. (b) The resistance changes of the G-foam in response to tensile, compressive and twisting strain. Reproduced with permission [115]. Copyright 2022, Elsevier. (c) Relative resistance changes of the organohydrogel electrode at various immersion times under 100% strain. (d) EMG signals recorded under different strains and (e) the corresponding SNR through the organohydrogel electrode. Reproduced with permission [112]. Copyright 2025, Wiley-VCH. (f) Schematic of the strain-insensitive meandering patterns of the ECG sensor, and (g) the analysis of stress distribution mapping using Von Mises simulation. Reproduced with permission [116]. Copyright 2024, Wiley-VCH. (h) Schematic illustration of the liquid metal-based kirigami electrode (LM-eKE) and (i) relative resistance (vertical axis) as a function of strain, with the blue line corresponding to LM-eKEs. Reproduced with permission [113]. Copyright 2022, Wiley-VCH. (For interpretation of the references to colour in this figure legend, the reader is referred to the Web version of this article.)

Deformation-insensitive materials represent a promising direction that leverages advanced composites and engineered microstructures to decouple electrical conductivity from mechanical strain. For instance, the G-foam electrodes, fabricated by coating porous polyurethane (PU) foam with vertically aligned gold nanowires (v-AuNWs), exhibits deformation-insensitive conductivity and impedance under substantial mechanical deformations—including tensile strains up to 40%, compression up to 80%, and twisting angles up to 1080° (Fig. 6a and b) [115]. The 3D porous architecture serves as a conformal, breathable skeleton that buffers mechanical deformation while the aligned nanowires maintain conductive pathways. The resultant G-foam electrodes can realize continuous, high stable ECG capture during various daily activities with an overall dynamic signal quality index (dSQI) > 0.85 for the continuous >3 h measurement. Similarly, lignosulfonate-derived conductive organohydrogel electrode demonstrates minimized MAs due to remarkable strain-insensitivity, with only 2-3% resistance change at 100% strain (Fig. 6c) [112]. This strain-insensitivity electrical behavior is attributed to following factors: salting-out-induced stretchable porous structures, mediated by the Hofmeister effect, can buffer mechanical deformation, while strong interactions between the polymer chains and ions stabilize the ionic conduction pathways during stretching. The resulting electrodes maintain stable signal amplitude and noise levels even under external vibrations, outperforming commercial Ag/AgCl electrodes in SNR (Fig. 6d and e). The resulting strain-insensitivity facilitates the acquisition of high SNR motion-artifact-free EP signals during skin or electrode deformation.

Engineered structures (*e.g.*, kirigami, serpentine, wavy structures) [84] offer alternative approaches to strain-insensitivity through structural designs that accommodate deformation without compromising electrical function. For example, a strain-insensitive breathable ECG electrode composed of carbon nanotube and nanoporous carbon (CNT@NPC) sensing elements integrated onto a hierarchical porous SEBS substrate was fabricated (Fig. 6f) [116]. The electrode's meandering and ripple-like structures design enables a wide range of detection under various stretching conditions, making it resistant to strains induced by skin deformations (0–15%) (Fig. 6g). The insensitivity to skin deformation effectively minimizes MAs. Besides, the CNT networks form railway-like conductive pathways that maintain electrical continuity under mechanical stress via tube-sliding mechanisms. This design ensures stable electrode-skin contact, showing high suppression characteristics to the strain and reduced signal distortion during human movements.

Besides, kirigami-patterned electrodes offer exceptional strain insensitivity [113,117]. An EGaIn-based elastic kirigami-patterned electrode (LM-eKE) exhibits minimal resistance changes (33% increase at 820% strain) during extreme deformation (Fig. 6h and i) [117], maintaining stable performance (<1.7% in resistance) change during joint movements like knee bending. The kirigami patterns achieve this by localizing strain to specific hinge regions while preserving consistent electrical connectivity under deformation. This electrical and mechanical stability enables reliable physiological monitoring (*e.g.*, EEG) without MA interference. However, the kirigami structures design of the electrodes may changes the electrode-skin contact area during deformation, leading to unstable signals [112].

**Core Material and Structural Design Commonalities of Strain-insensitive Strategy**: The deformation constraint strategy is based on the core design principle of decoupling electrical conductivity from mechanical strain. The representative materials/structures share the following key features:

i) Core material properties

**Deformation-insensitive conductivity**: The resistance/impedance of conductive materials change rate is very small under large strain.

**High mechanical deformability**: Tolerating large stretching/compressive/twisting strain.

ii) Structural design commonalities

**Engineered structures**: Kirigami, serpentine, wavy, wrinkle or meandering structures, which localize strain to specific hinge regions and protect the conductive sensing area from deformation.

**Patterned conductive pathways**: Serpentine, wavy and other conductive pathways exhibit significant strain-insensitive behavior until they are fully straightened or their geometric deformation limits are reached.

Balancing strain insensitivity with electrode-skin contact area, some engineered structures (*e.g.*, kirigami) or patterned conductive pathways may cause changes in contact area during deformation, which needs to be optimized by structural patterning to ensure stable contact.

### 4.4 Damping strategy

The damping strategy is targeted at solving interfacial impedance instability, potential instability and even friction that caused by low-frequency mechanical energy (overlaps with the frequency band of EP signals (0.1–30 Hz)). This strategy absorbs or dissipates the dynamic mechanical energy generated by body movements (*e.g.*, respiration (0.1-1 Hz), walking (1-2 Hz), limb swinging (10-15 Hz)) by using viscoelastic materials or damping microstructures, and attenuates the mechanical stress transmitted to the electrode-skin interface, thus avoiding the impedance instability and biopotential instability caused by mechanical disturbance. Damping strategy offers unique advantages for dynamic recording environments where post-processing filtering techniques face fundamental limitations in separating overlapping EP signals and MA frequencies. However, this strategy has received little attention but represents a promising solution for suppressing MAs.

**Fig. 7:**
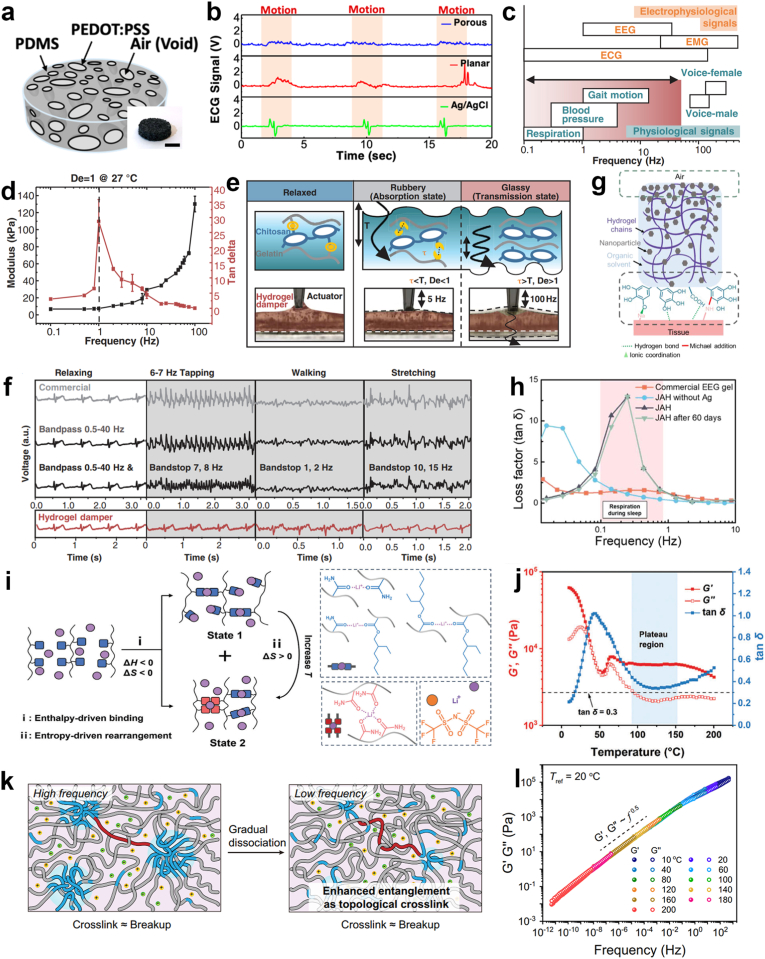
Damping strategy. (a) Photograph and schematic of the sponge electrode. (b) Compare of ECG signals recorded in the presence of motions caused by periodic body movements. Reproduced with permission [55]. Copyright 2022, American Chemical Society. (c) Typical frequency ranges for human-related movements and electrophysiological signals. (d) Dynamic storage modulus and tan δ of the gelatin-chitosan hydrogel as a function of frequency at the temperature range of 27 °C. (e) The selective damping mechanism in the hydrogel damper is linked to the relaxation time. The transition between absorption and transmission is influenced by the Deborah number (De) of the hydrogel damper (upper). The hydrogel damper placed on a 1.5 mm polyethylene terephthalate (PET) substrate exhibits fluctuations under a 100-Hz vibration, transmitting vibrations when De > 1 (lower). (f) ECG signals recorded using a commercially available 3M electrode, followed by bandpass filtering (0.5 to 40 Hz), additional bandstop filtering with different ranges (top) and (bottom) the hydrogel damper under mechanical noise including tapping, breathing, and walking. Reproduced with permission [10]. Copyright 2022, AAAS. (g) The mechanism of asymmetric adhesion of JAH. (h) Loss factors of the JAH, JAH without Ag, JAH after 60 days, and a commercial EEG gel as a function of frequency. Reproduced with permission [118]. Copyright 2024, Springer Nature. (i) Thermodynamic explanation of the enthalpy-driven binding process in SICE and the entropy-driven rearrangement of dynamic ion-dipole interactions. Heating causes low-coordinated ion-dipole interactions (State 1) to release Li^+^, resulting in high-coordinated ion-dipole interactions (State 2), which enhances effective cross-linking and thermal stability of the SICE. (j) Rheological results obtained through temperature sweeping of SICE. Reproduced with permission [119]. Copyright 2024, Wiley-VCH. (k) As frequency decreases, physical interactions gradually dissociate, leading to simultaneous interchain breakup and increased entanglement acting as topological crosslinks. (l) Rheological master curves of the P(BA-co-MAA) ionogel with the critical gel point state across an ultra-wide frequency range. Reproduced with permission [120]. Copyright 2024, Springer Nature.

Microstructural-based damping approaches utilize damping microstructures to absorb mechanical energy and minimize relative displacement between electrodes and skin. For instance, an elastomeric PDMS/PEDOT:PSS sponge electrode with conductive hydrogels embedded in its micropores was developed (Fig. 7a) [55]. The sponge electrode's soft and porous structure acts as a shock absorber that minimizes relative displacement between the electrode and the skin. Simultaneously, the gel-filled micropores could also serve as an interfacial buffer layer that maintains a relatively constant contact area and balances interfacial charge fluctuations during motions. Benefiting from these properties, this sponge electrode demonstrates robust high-quality EP signals acquisition with mitigated MAs during physical activity, outperforming conventional planar and Ag/AgCl electrodes in motion-artifact tolerance (Fig. 7b).

Material-based damping approaches leverage the intrinsic viscoelastic properties of viscoelastic materials. These systems dissipate mechanical energy through reversible breakage of dynamic bonds (*e.g.*, hydrogen bonds, disulfide bonds, B–O/B–N dynamic bonds) and internal/intermolecular molecular frictions [15,121]. The materials’ damping capacity is quantified by the loss factor (tan δ = G''/G′), defined as the ratio of the loss modulus (G″) to the storage modulus (G′) [122]. A viscous-dominated response (tan δ > 1) results in efficient conversion of mechanical energy into heat, whereas tan δ < 1 indicates a predominantly elastic behavior [123]. The employment of dynamic bonds commonly increases tan δ. Compared to conventional elastomers (*e.g.*, PDMS) with low loss factors (tan δ < 0.1), materials with a high tan δ value (>0.5) demonstrate superior energy dissipation capacity, making them particularly suitable for damping applications in dynamic environments. Crucially, these viscoelastic materials can be engineered for selective frequency damping, particularly targeting common physical activity frequencies (*e.g.*, respiration at 0.1-1 Hz, walking at 1-2 Hz, limb swinging at 10-15 Hz, heart beating at 0.3‒4 Hz) that overlap with key EP signals (Fig. 7c) [10,119].

To specifically attenuate MAs, it is essential to first identify the target center frequency or the specific frequency range requiring suppression. For a dynamic polymer network with a single sharp tan δ peak (G′-G″ crossover angular frequency, ω_c_ = 2π*f*), the frequency at which this peak occurs needs to be strategically aligned with the target MA frequency to maximize damping performance. The inverse of ω_c_ corresponds to the relaxation time τ in ideal Maxwell-type materials.

(4.3) $$\tau =\frac{1}{\omega_{c}}$$

To achieve this target τ, the segmental dynamics of the network can be tuned through several strategies including adjusting cross-linking density, selecting dynamic bonds with appropriate strength and incorporating plasticizers. Typically, a reduction in cross-linking density enhances the mobility of polymer chains, leading to a decrease in the average τ. The addition of plasticizers increases chain segment mobility, effectively shortening the average τ. To effectively dampen a broad frequency range rather than a single frequency, the distribution of relaxation times must be broadened, resulting in broadened or multi-peaked tan δ spectrum. The broadened spectrum reflects a wide range of dynamic processes. This can be achieved by introducing structural heterogeneities such as phase separation or multi-scale dynamic cross-linking designs. Aggregates with varying sizes possess different binding strengths, which correspond to distinct relaxation times, resulting in a spectrum of τ values. Similarly, incorporating multiple distinct dynamic bonds may display multiple dissipation modes, with two or more tan δ peaks appearing across frequency ranges [123]. Thus, the broad distribution manifests as a widened tan δ peak or maintains a high tan δ value across a wide frequency range, enabling effective damping over the desired range. Networks with greater dynamicity tend to exhibit a lower ω_c_, higher tan δ peak values, and broader tan δ peaks, reflecting faster and more distributed bond exchange processes [123].

MAs caused by low-frequency movements (<30 Hz), significantly degrade EP signals monitoring. Traditional bandpass filters reduce noise but cause signal distortion. Inspired by the frequency-selective vibration damping capability of spider cuticular pads that separate target signals from the noisy environments (*i.e.*, windy, rainy, the presence of prey), a viscoelastic gelatin-chitosan interpenetrating hydrogel electrode with shear-thickening property was proposed (Fig. 7d) [10]. This hydrogel exhibits a frequency-dependent phase transition: at low frequencies (<30 Hz), it behaves as a rubbery state to absorb low frequency dynamic mechanical noise, while at higher frequencies (>30 Hz), it transitions to a glassy state to transmit target EP signals with an increased modulus (Fig. 7e). This behavior is governed by the Deborah number (De = relaxation time/vibration period). When De < 1, weak bonds (*e.g.*, hydrogen bonds) break to dissipate low-frequency energy; when De > 1, the bonds cannot reorganize rapidly enough, enabling high-frequency signal transmission with minimal attenuation. Besides, the transition frequency is determined by the relaxation time of the viscoelastic hydrogel and temperature. The hydrogel electrode shows stable EP monitoring even under a variety of activities owing to its selectively eliminate dynamic mechanical noise (Fig. 7f). Similarly, a Janus adhesive hydrogel (JAH) with selective frequency damping capability was developed for MA-resistant bioelectronic interfaces [118], which consists of a polyacrylamide matrix embedding with gradient-distributed silver nanoparticles (Fig. 7g). The JAH enables selectively damping dynamic motions within the respiratory frequency range (0.1–1 Hz). The loss factor within this range is 60 times higher than that at other frequencies (Fig. 7h). The frequency selectivity stems from the JAH's internal energy dissipating bonding design. Under respiration-induced dynamic noise, weak bonds within the JAH dissipate energy through reversible breaking, while strong ionic coordination and hydrogen bonds at the interface maintains stable adhesion and low contact impedance. This dual-function design effectively suppresses MAs, ensuring high-fidelity EP signals monitoring. However, dynamic bonds within the damping electrodes are susceptible to dissociation as the temperature increasing, leading to a rapid decline in the loss factor (tan δ) and a consequent narrowing of the effective damping temperature range.

Supramolecular ion-conductive elastomers (SICE) combining weak-to-strong ion-dipole interactions also demonstrate impressive damping performance and mechanical properties across an ultra-wide temperature range (18-200 °C) [119]. The SICE, composed of acrylamide (AAm), 2-ethylhexyl acrylate (EHA), and lithium bis(trifluoromethanesulfonyl)imide (LiTFSI), leverages synergistic enthalpy-entropy balancing: strong ion-dipole interactions enhance the network strength by a high enthalpy gain, while dynamically weaker ion-dipole interactions are responsible for energy dissipation through reversible bond breaking (Fig. 7i). Crucially, entropy-driven rearrangement of Li^+^–O ion-dipole interactions stabilize damping properties over temperature extremes (Fig. 7j). The SICE shows a damping capacity of 91.2% and a peak tan δ of 1.11 by dissipating vibrational energy to heat through controlled ion-dipole interactions breakage. By decoupling ionic conduction (soft phase) from energy dissipation (dynamic bonds), the design maintains stable electrode-skin impedance during movement, enabling low MAs EP monitoring under different dynamic mechanical interference. The damping and mechanical properties of SICE can be tailored by precise modulation of ion-dipole interaction strength, offering a versatile platform for MA-resistant wearable electrodes.

Different from the traditional ionic skins dominated by either viscosity or elasticity at most frequencies and have only one single gel point at a fixed frequency, a poly(butyl acrylate-*co*-methacrylic acid) associative polymer self-compliant ionic skin works consistently at the critical gel point state (tan δ = G′′/G′ ≈ 1) with almost equal viscosity and elasticity across an unprecedented ultra-wide frequency range (10^−11^–500 Hz) (Fig. 7l) [120]. By taking advantage of this gel point state, the ionic skin not only dissipates energy for dynamic compliance, but also durably recovers its shape to prevent free flow. This sustained equilibrium between interchain crosslink and breakup is achieved through a hierarchical hydrogen bond association design with phase-separated nanostructure, which broadens the distribution of physical interaction binding strengths. As the frequency decreases, gradual chain dissociation occurs, as the larger aggregates are more resistant to dissociation and tend to remain intact. Through careful tuning of the associative group density and the phase-separated structure of the supramolecular network, the complementary effect between associative and entanglement crosslinks ensures the material remains consistently at the gel point state, helping to sustain the balance between viscosity and elasticity (Fig. 7k). The combined attributes of self-compliance, softness, adhesiveness, stretchability, self-healing, and flaw insensitivity emphasize the superiority of the ionogel electrode over conventional viscous or elastic ionic skins for dynamic conditions, which was demonstrated for high-fidelity ECG and sEMG monitoring with reduced vibration interference and minimized MAs.

**Core Material and Structural Design Commonalities of Damping Strategy**: The damping strategy is based on the core design principle of frequency-related energy dissipation. The representative materials/structures share the following key features:

i) Core material properties

**Dynamic bond networks**: Hydrogen bonds, ion-dipole interactions or disulfide bonds break reversibly to dissipate mechanical energy.

**High low-frequency loss factor (tan δ)**: high tan δ > 0.5 in the low-frequency band (0.1–30 Hz) can effectively dissipate mechanical energy caused by body movements.

ii) Structural design commonalities

**Damping microstructures**: Porous/sponge structures can act as shock absorbers to minimize electrode-skin relative displacement.

### 4.5 Strategy comparations

The four primary strategies discussed in the previous sections for MA suppression each address distinct aspects with relative strengths and limitations, these strategies are not isolated from each other. This section aims to clarify the logical hierarchy and complementary relationships among these strategies. Based on their operating mechanisms, their suitability for different application scenarios and for recording various EP signals will be analyzed.

These four strategies can be organized into a clear, logically MA suppression framework:

1) **Interface Motion Management.** It directly addresses the mechanical mismatch at the electrode-skin interface, serving as the basic suppression against MAs. The skin-anchoring strategy adopts a philosophy of “motion following.” It uses strong anchoring forces to create a mechanical union between the electrode and the skin, minimizing relative slippage and thereby stabilizing the contact impedance and reducing friction. In a complementary approach, the deformation constraint strategy employs a philosophy of “motion isolation”. It uses locally rigid structures or even clever angled embedding to establish a mechanical isolation zone or decoupling region between the sensing area and the deforming skin. This prevents large skin stretches or compressions from being effectively transmitted to the sensing area of the electrode.
2) **Electrode Performance Optimization.** When mechanical disturbances inevitably reach the electrode itself, the strain-insensitive strategy and damping strategy come into play. The strain-insensitive strategy ensures that the conductive pathways or sensing units of the electrode maintain stable electrical properties under deformation such as stretching or bending, thereby decoupling mechanical strain from electrical signal modulation. This is typically achieved using intrinsically stretchable conductors (*e.g.*, liquid metals) or carefully designed engineered topologies (*e.g.*, kirigami structures). Besides, damping strategy utilizes the energy dissipation properties of viscoelastic materials or microstructures to convert mechanical kinetic energy, particularly in frequency bands that overlap with MAs (often low frequencies) into heat. This acts as a “filtering” attenuation of the mechanical energy.

In brief, the former (interface motion management) addresses the mechanical mismatch at the electrode-skin interface, while the latter (electrode performance optimization) ensures the stability of the electrode's performance when such disturbances are inevitably transmitted. However, a single strategy is difficult to deal with all dynamic challenges. It seems that the most effective designs for motion-artifact-resistant electrodes often require the incorporation of these strategies. For instance, an ideal electrode for dynamic monitoring might simultaneously feature: a strongly adhesive and conformal contact with the skin (skin-anchoring strategy), a strain-isolating structure that protects core sensing areas (deformation constraint strategy), stable electric performance under strain (strain-insensitive strategy), and a substrate material that absorbs low-frequency vibrations (damping strategy).

The overview of source-level MA suppression strategies and their performance is given in Table 3. The optimal strategy selection may depend on multiple factors including the EP signals type being recorded, the movement amplitude/intensity, and manufacturing constraints. The optimal application scenario for each strategy is as follows: Skin-anchoring strategy is optimal for low-to-medium intensity dynamic scenarios (*e.g.*, daily walking, office ambulatory monitoring), which is most suitable for monitoring sites with relatively flat skin and small deformation. It is the basic and most widely applicable strategy for all EP signals (serving as a foundational design for combined strategies). Deformation constraint strategy is optimal for ECG/sEMG in medium-to-intensity dynamic scenarios (*e.g.*, jogging, swimming), which is most suitable for monitoring sites with large-area skin deformation (chest, abdominal, large muscle groups of arms/legs). It is the first choice for suppressing MA caused by skin stretching/compression, and is particularly effective for ECG monitoring with obvious chest deformation during breathing/exercise. Strain-insensitive strategy is optimal for sEMG in high-intensity dynamic scenarios (sprinting, ball games, joint flexion/extension), which has advantage for monitoring sites with extreme local deformation. It is the only strategy that can effectively suppress MA caused by electrode resistance changes under large strain, and its application in ECG/EOG/EEG does seem to have some effect, but the effect appears to be rather limited. Damping strategy proves to be particularly effective for all EP signals in low-to-medium intensity dynamic scenarios, making it most suitable for monitoring sites that require high signal sensitivity and are dominated by low-frequency mechanical noise. Its unique frequency-selective damping capability solves the problem of signal distortion caused by traditional post-processing filtering (MA and EP signal frequency complete overlap), and it is the irreplaceable strategy for low-frequency MA-dominated EP signals monitoring. In brief, for static/low-intensity daily monitoring, the skin-anchoring strategy is typically a fundamental and efficient choice, offering significant stability enhancement with relatively low realization complexity. For high-intensity/large-amplitude motion or professional sports monitoring, deformation constraint, strain-insensitive, and damping strategies appear to be more effective options. A combined strategy may be worth considering but is currently rarely reported, such as employing both deformation constraint and damping strategies to address local deformation and residual energy dissipation.

**Table 3:** Overview of source-level strategies and their performance in suppressing MAs.

Strategy	Method	Material & Structure	Target signal	Interface impedance	SNR [dB]	Electrode location/Motion type corresponding to SNR	SNR Normalization [dB]	Ref.
Skin-anchoring strategy	Interfacial adhesion enhancement	CNT/graphene/PDMS & Gecko-like structures	ECG	/	/	Left & right wrists/Wrist curl, squat, writing	/	[82]
		PDMS/rGO & Hexagonal micropatterns/Octopus-like convex cups	ECG/sEMG/EEG	/	/	Forearm/Hand movements, flowing water (sEMG)	/	[83]
		PAA-Ag-SEBS/EGaIn- sodium alginate textile	ECG/EOG/EEG	/	12.8 ± 1.6 (ECG) 12.2/17.5 (EEG)	Clavicle/Rope skipping (ECG) Forehead/Eyes close and open (EEG)	/	[2]
	Conformality enhancement	Au/Cr/PET & Serpentine shape	ECG	/	/	Chest/Skin indentation, chest expansion	/	[84]
		Graphene/PEDOT:PSS	ECG/sEMG/EOG/EEG	32 kΩ (@100 Hz) 45 kΩ (Ag/AgCl @100 Hz)	21 ± 0.7 (sEMG) 14 ± 0.4 (Ag/AgCl@ sEMG)	Forearm/Electromechanical vibrator (sEMG)	∼7 (sEMG)	[36]
		PAA/chitosan/MXene	ECG/sEMG	/	28.0 (sEMG) 25.3 (Ag/AgCl@ sEMG)	Forearm/Hand movements (sEMG)	2.7 (sEMG)	[85]
		Parylene C-polyacrylamide/polypyrrole/gelatin hydrogel	ECG/sEMG	/	46.6/19.8 (ECG/sEMG) 41.6/0.7 (Ag/AgCl@ECG/sEMG)	Chest/Poking with glass rod (ECG) Forearm/Running (sEMG)	5 (ECG) 19.1 (sEMG)	[86]
		Cotton-PVA/TA hydrogel	ECG	/	37.8 (ECG) 20.6 (Ag/AgCl)	Forearm/Wrist bending	17.2 (ECG)	[87]
		Polyborosiloxane/D-sorbitol/conductive carbon filler	ECG/sEMG/EEG	132 ± 23 kΩ•cm^2^(@100 Hz)	38/40/1 (ECG@0.2/0.5/1 Hz)	Chest/Chest expansion (ECG)	/	[124]
	Friction enhancement	Ag/AgCl/TPU & Fructus xanthii-inspired barbed structure	ECG	31.6/27.6 kΩ (@0.1Hz/10 Hz) 21.4/20.7 kΩ (Ag/AgCl @0.1Hz/10 Hz)	27.77 (ECG) 21.92 (Ag/AgCl@ECG)	Left & right chest/Arm swinging	5.85 (ECG)	[88]
	Contact pressure improvement	MXene/CNF/polycarboxylate ether/TPU-laminated cotton fabric	ECG/sEMG	191 kΩ (@100 kHz) 64 kΩ (Ag/AgCl @100 kHz)	/	Chest/Walking, driving (ECG)	/	[106]
Deformation constraint strategy	Stress/strain isolating	Silver-coated yarn textile & Support cushion structure	ECG	/	/	Forearm/Servo motions	/	[72]
		Au/Cr/PI/PDMS & Polypropylene strain-isolator structure	ECG	/	21.6 (ECG) 12.1 (MAX-ECG@ECG)	Chest/Jogging	9.5 (ECG)	[5]
	Island-bridge	Au/Cr/PI island-bridge structure	ECG	0.94/0.39 MΩ (@30/100 Hz)	∼40 (ECG) 45.3 (Ag/AgCl)	Chest/Running	−5.3 (ECG)	[107]
Strain-insensitive strategy	Deformation-insensitive material design	Vertically aligned AuNWs/PU foam	ECG	175.7 ± 7.6 Ω (@1 kHz)	/	Chest/Walking, running, jogging	/	[115]
		Polyacrylamide/lignosulfonate/silk fibroin organohydrogel	ECG/sEMG	5.2 kΩ (@1 kHz) 6.3 kΩ (Ag/AgCl @1 k Hz)	34.5/21.53 (ECG/sEMG) 25.61/19.01 (Ag/AgCl@ECG/sEMG)	Chest/In static (ECG) Forearm/Clenching, gripping at increasing force (sEMG)	8.89 (ECG) 2.52 (sEMG)	[112]
	Deformation-insensitive structural design	Liquid metal/Au/silicone & Kirigami structure	EEG	/	/	Forehead/Eyes close and open (EEG)	/	[117]
		CNT/nanoporous carbon/hierarchical porous SEBS & Meandering and ripple-like structures	ECG	/	26 ± 1/19.5 ± 3.4 (0/15% strain)	Forearm and finger/Stretching	/	[116]
		PVA/aramid nanofiber/PEDOT & Kirigami structure	ECG/sEMG	120-180 kΩ (@100 Hz)	/	Chest/In static (ECG)	/	[113]
Damping strategy	Structural-based damping	PEDOT:PSS/PDMS sponge/conductive electrolyte gel	ECG/sEMG	12.2 kΩ (@10 Hz) 80.4 kΩ (Ag/AgCl @10 Hz)	23.1 (ECG) 22.6 (Ag/AgCl@ECG)	Left and right arms/Rapidly standing up & sitting down (ECG)	0.5 (ECG)	[55]
	Viscoelastic material-based damping	Gelatin/chitosan hydrogel	ECG/EEG	/	/	Chest/Tapping, breathing, walking and stretching (ECG) Forehead/Actuator (EEG)	/	[10]
		Polyacrylamide/TA/NaCl/AgNPs Janus hydrogel	ECG/EOG/EEG	/	/	Chest/Breathing (ECG) Canthus/Breathing (EOG)	/	[118]
		Polyacrylamide/EHA/LiTFSI	ECG/sEMG	/	/	Chest/Tapping, walking and stretching (ECG) Arm/Tapping (sEMG)	/	[119]
		P(BA-co-MAA)/liquid metal ionogel	ECG/sEMG	/	/	Left and right wrists/Vibrating, walking, running (ECG) Forearm/Vibrating by vibrating ball (sEMG)	/	[120]

*SNR Normalization:* the ratio of the SNR measured by the designed electrode to that of the gold-standard Ag/AgCl electrode.

However, due to variations in individual skin conditions, measurement locations, and SNR calculation methods across different literature, the calculated SNR values appear to have a certain degree of incomparability. Even when using commercial Ag/AgCl electrodes, the SNR values given by different literature can still show significant differences. Therefore, a more reasonable approach for comparison might be to directly contrast the normalized SNR (*i.e.*, the SNR of the proposed electrode relative to that of a standard Ag/AgCl electrode under identical test conditions) and examine the measured EP signals. This approach may be more intuitive and can help eliminate the influence of a series of irrelevant factors.

## 5 Challenges and future perspectives

Despite significant advances in source-level MA suppression, the complete elimination remains an ongoing challenge. The strategies discussed—skin-anchoring, deformation constraint, strain-insensitive and damping strategies—have demonstrated considerable efficacy in stabilizing the skin-electrode interface. However, each strategy individually is often insufficient to deal with the multifaceted and unpredictable nature of dynamic real-world environments. These dynamic real-world environments introduce complexities, such as varying physiological states, sustained mechanical stress, and diverse user activities, which rarely encountered in controlled laboratory settings. While the most direct path forward appears to lie in the synergistic integration of the aforementioned strategies, the translation of such integrated designs from laboratory prototypes to reliable, real-world applications continues to face significant hurdles. Overcoming these barriers will require continued material innovation, sophisticated engineering solutions, and validation in truly dynamic real-world environments.

A foundational step towards this goal is gaining a comprehensive understanding of the underlying mechanisms of MA generation, as it provides the fundamental rationale for designing robust, MA-free electrodes. Besides, developing standardized MA benchmarks can provide a more intuitive and effective comparison of the MAs suppression effects among different literature. It might be possible to achieve this by using standardized SNR or interface impedance. (*i*.*e*., the SNR or interface impedance of the proposed electrode relative to that of a standard Ag/AgCl electrode under identical test conditions).

A primary challenge lies in achieving reliable performance under complex and dynamically evolving physiological conditions. Human skin undergoes continuously natural processes including perspiration, hair growing, sebum production, and the regeneration of the stratum corneum (approximately every 24 h). These processes can degrade the contact integrity of electrodes: perspiration creates unpredictable ionic pathways and promotes delamination; lipids form insulating layers that increase impedance; hair physically obstructs contact; and stratum corneum increases interfacial contact impedance [4,31,125,126]. Such degradation potentially disrupts the skin-electrode interface and exacerbates its instability during body movements, resulting in increased MAs and signal inaccuracy. Critically, these factors do not act in isolation but may interact over time, making long-term stability a distinct and more demanding challenge than short-term performance.

In response, recent research has focused on developing breathable electrodes and sensing platforms compatible with skin's natural barrier function [2,22,127], with this function often being assessed by transepidermal water loss (TEWL). TEWL values below 10-15 g/(m^2^·h) typically indicate intact skin, while higher values suggest compromised barrier function [128]. Electrodes that occlude the skin can artificially elevate local TEWL, leading to moisture accumulation, skin irritation, and unstable contact. Thus, high breathable and sweat permeable electrodes that are compatible with TEWL can prevent sweat accumulation and the resulting MAs, while maintaining physiological TEWL microenvironment at the electrode-skin interface for stable long-term monitoring. Furthermore, adhesion under wet or underwater conditions is also a factor that needs to be considered for real-world scenarios (*e.g.*, during intense exercise or swimming), as perspiration or moisture can compromise electrode adhesion or even cause detachment.

The dynamic evolution of the skin-electrode interface over extended periods (hours to days) reveals a critical distinction between initial and long-term performance. While skin abrasion yields the lowest initial interfacial impedance temporarily, the impedance of both abraded and untreated skin gradually converges several hours later and stabilizes at the same level owing to stratum corneum regeneration. This short-term strategy focused on drastically lowering initial impedance are impractical and unnecessary for long-term applications, as they raise concerns about skin barrier damage and infection risk [74,79]. Instead, the goal for long-term performance shifts to achieving a stable and equilibrium interface that can withstand the skin's natural physiological process. Therefore, future electrode innovations must integrate these considerations comprehensively. The objective should shift from merely resisting physiological challenges to actively harmonizing with the skin's long-term dynamic state. This approach is crucial for enabling practical, real-world deployment of the electrodes.

Beyond the interface, cable and connector management presents another persistent source of MAs that receives insufficient attention [58,74]. The triboelectric effect from cable movement generates significant MAs that causes charge accumulating on the surfaces of the cables [71,76]. This occurs as friction and deformation between adjacent cable insulation layers modify the electrostatic voltage, which is influenced by factors including the material properties of the cables, the contact area, the type of contact, and the speed of relative displacement [71]. Moreover, the stretching of the electrode connector may lead to the sliding between the electrode and the connector. A stretchable connector can helps reduce MA noise during movements that stretch the entire device [58]. However, these stretchable cables and connectors still appear to be relatively fragile and the triboelectric effect may still exist. There remains a need to develop more effective methods to reduce MAs that may be caused by cables and connectors.

A holistic co-design strategy is indeed crucial for effective MA management in wearable electrophysiology. While this review focuses on source-level MA suppression at the electrode-skin interface, its ultimate goal is to provide cleaner signals to the downstream circuitry and algorithms. Thus, the analog front-end (AFE) must be specifically tailored to interface with these advanced electrodes. For instance, to accommodate the high and variable impedance characteristic of dry electrodes, the AFE requires dedicated design, such as ultra-high input impedance, DC offset cancellation and enhanced common-mode rejection rate (CMRR). For in-band specific frequency MAs, circuit-level techniques like chopping, notching or analog filters can provide initial, low-power suppression before digitization. Furthermore, through hardware strategies such as signal compensation circuits that utilize differential acquisition and compensation channels to subtract artifact signals, MAs can be targeted for mitigation regardless of their features. These electrode-hardware co-designs actively shape the signal quality before digitization, reducing the burden on subsequent post-processing algorithmic intervention. Algorithmic intervention employs techniques such as filtering (*e.g.*, Butterworth, wavelet transform) for removing unwanted frequency components, and AI models for correction and suppressing MAs from complex EP signals. However, a fundamental challenge in wearable system design lies in balancing performance with stringent power consumption and size constraints. Complex AFE circuits and algorithms increase power and area overhead, while simply streaming raw data for cloud processing incurs latency, privacy concerns, and high continuous communication energy costs. Thus, there are difficult trade-offs between AFE complexity, the computational capacity/power of local embedded processors, and the demands of wireless transmission. For latency-critical applications, a co-designed system would embed a quantized, lightweight AI model directly into the wearable's low-power processor, enabling real-time inference at the edge. This approach balances analysis accuracy with the system's real-time and power constraints. In summary, this hierarchical co-design philosophy—encompassing electrode design, intelligent circuitry, and embedded algorithms—establishes a synergistic framework where each level augments the others, thereby paving the way for MA-robust, efficient, and clinically viable wearable bioelectronic systems.

Looking forward, future advancements will converge toward “intelligent MA-free electrodes" that actively adapt to dynamic physiological and broad real-world scenarios. The most transformative opportunity for next-generation electrodes in MA suppression may lies in the intelligent co-design of materials and structures with dynamic properties through artificial intelligence (AI) and machine learning [129]. The multidimensional optimization space encompassing mechanical properties, electrical characteristics, interfacial behavior, and manufacturing parameters exceeds the capabilities of traditional trial-and-error approaches. AI algorithms can rapidly identify non-intuitive design rules and predict optimal configurations for specific application scenarios. Generative models can propose novel material compositions with tailored viscoelastic and conductive properties, while neural network-enhanced multiphysics simulations can accurately model dynamic interface behavior under complex mechanical stresses.

Future wearable electrode systems may integrate real-time impedance monitoring with closed-loop adjustment of interfacial properties, multimodal sensor fusion combining mechanical (*e.g.*, strain, pressure) and electrical data for adaptive MA cancellation, biomimetic designs that replicate the sophisticated damping and adhesion mechanisms found in natural systems, and soft robotics-inspired interfaces with tunable stiffness and adhesion that enabling dynamic optimization for interfacial stability and comfort during movement. Success will depend on collaborative efforts across disciplines including materials science, mechanical engineering, electrical engineering, and computer science. By synergistically addressing MA origins through materials, mechanics, and intelligence, next-generation MA-free electrodes will achieve reliable monitoring in dynamic environments, unlocking transformative potential in clinical diagnostics and personalized health tracking.

## CRediT authorship contribution statement

**Haizhou Huang:** Conceptualization, Formal analysis, Funding acquisition, Project administration, Supervision, Visualization, Writing – original draft, Writing – review & editing. **Xuanjing Cai:** Visualization, Writing – original draft. **Shu Wan:** Funding acquisition, Supervision, Writing – review & editing. **Litao Sun:** Funding acquisition, Supervision, Writing – review & editing.

## Declaration of competing interest

The authors declare that they have no known competing financial interests or personal relationships that could have appeared to influence the work reported in this paper.

## Data Availability

Data will be made available on request.
